# Twenty-seven-nucleotide repeat insertion in the *rplV* gene confers specific resistance to macrolide antibiotics in *Staphylococcus aureus*

**DOI:** 10.18632/oncotarget.25441

**Published:** 2018-05-25

**Authors:** Dianpeng Han, Yu Liu, Jingjing Li, Chenghua Liu, Yaping Gao, Jiannan Feng, Huizhe Lu, Guang Yang

**Affiliations:** ^1^ Beijing Institute of Basic Medical Sciences, Beijing, China; ^2^ State Key Laboratory of Toxicology and Medical Countermeasures, Beijing, China; ^3^ Henan University School of Basic Medical Science, Kaifeng, China; ^4^ Department of Applied Chemistry, College of Science, China Agricultural University, Beijing, China

**Keywords:** macrolides resistant, rplV, repeat insertion, Staphylococcus aureus

## Abstract

Macrolide antibiotics are used for treatment of soft-tissue infection caused by *Staphylococcus aureus* in humans. However, infections with *S. aureus* are increasingly difficult to treat owing to the emergence and rapid spread of multiple-drug resistant *S. aureus*. Resistance to macrolide in *S. aureus* is mostly due to the modification of 23 S rRNA by methylases encoded by *erm* genes. Here, we have identified that a 27-nucleotide repeat sequence insertion in the *rplV* gene induced a specific resistance to macrolide antibiotics. An erythromycin-resistant strain, 8325^ER+^, was screened by resistance to erythromycin from the macrolide-sensitive strain 8325-4. Comparative genome sequencing analysis showed that 8325^ER+^ contained a 27-nt repeat sequence insertion in the *rplV* gene that encodes the ribosomal protein L22, when compared to its parent strain. The 27-nt repeat sequence led to an insertion of 9 amino acids in L22, which had been identified to reduce the sensitivity to erythromycin and other macrolide antibiotics. Moreover, we show that the ectopic expression of the mutated *rplV* gene containing the 27-nt repeat sequence insertion in several susceptible strains specifically conferred resistance to macrolide antibiotics. Our findings present a potential mechanism of resistance to macrolide antibiotics in *S. aureus.*

## INTRODUCTION

*Staphylococcus aureus* is the leading Gram-positive bacterium that can cause infections in humans worldwide, including mild skin infections, bacteremia, sepsis, and endocarditis [1–3]. Over the last century, infections with *S. aureus* have become increasingly difficult to treat owing to the emergence and rapid spread of multiple-drug resistant *S. aureus* [4–6].

Macrolides, which consist of a 14- to 16-membered lactone ring with different appended sugars and comprise a key group of inhibitors of bacterial translation, are ribosome-targeting antibiotics used to treat infections caused by *Staphylococcus* species [7, 8]. Erythromycin, azithromycin, and clarithromycin are members of the macrolide antibiotics, a large group of antibacterial agents that include natural or newer semi-synthetic compounds [9, 10]. Their inhibitory activity depends on binding to a site near the ribosomal nascent peptide exit tunnel, which starts at the peptidyl transferase center and spans the body of the large ribosomal subunit, thereby halting translation of a particular subset of nascent peptides [11–13].

Resistance to macrolide may be mediated by three primary mechanisms: a) modification of ribosomes, such as dimethylation of a unique adenine residue in the 23S ribosomal RNA (rRNA), A2085 in *S. aureus* (corresponding to *E. coli* A2058), which is located in the macrolide-binding site in the nascent peptide exit tunnel, by the erythromycin resistance methyltransferase encoded by the *erm* genes [14–16]; b) activated efflux systems, involving a member of the ATP-binding cassette (ABC) family of transporters encoded by the macrolide-streptogramins resistance A (*msrA*) gene, keeping intracellular antibiotic concentration at a subtoxic level and conferring inducible resistance to erythromycin and type B streptogramins in staphylococci [17, 18]; and c) production of antibiotic-inactivating enzymes, such as phosphorylase, a macrolide phosphotransferase C (encoded by *mphC* in staphylococci) that inactivates antibiotics [19, 20]. According to other studies, mutations in *Escherichia coli rplV* and *rplD* genes coding for ribosomal proteins L22 and L4, respectively, can also confer resistance to macrolide antibiotics [21, 22]. A mutant change in *rplV* was also observed in antibiotic-resistant *S. aureus* [23].

Here, we screened a resistant strain obtained by culturing the sensitive *S. aureus* strain 8325-4 in the presence of erythromycin. A 27-nt repeat sequence insertion in the *rplV* (*rplV^indel^*) gene was identified in this erythromycin-resistant strain, which induced specific resistance to macrolides.

## RESULTS

### An erythromycin-resistant strain of *S. aureus* 8325-4 is screened *in vitro*

**Figure 1:**
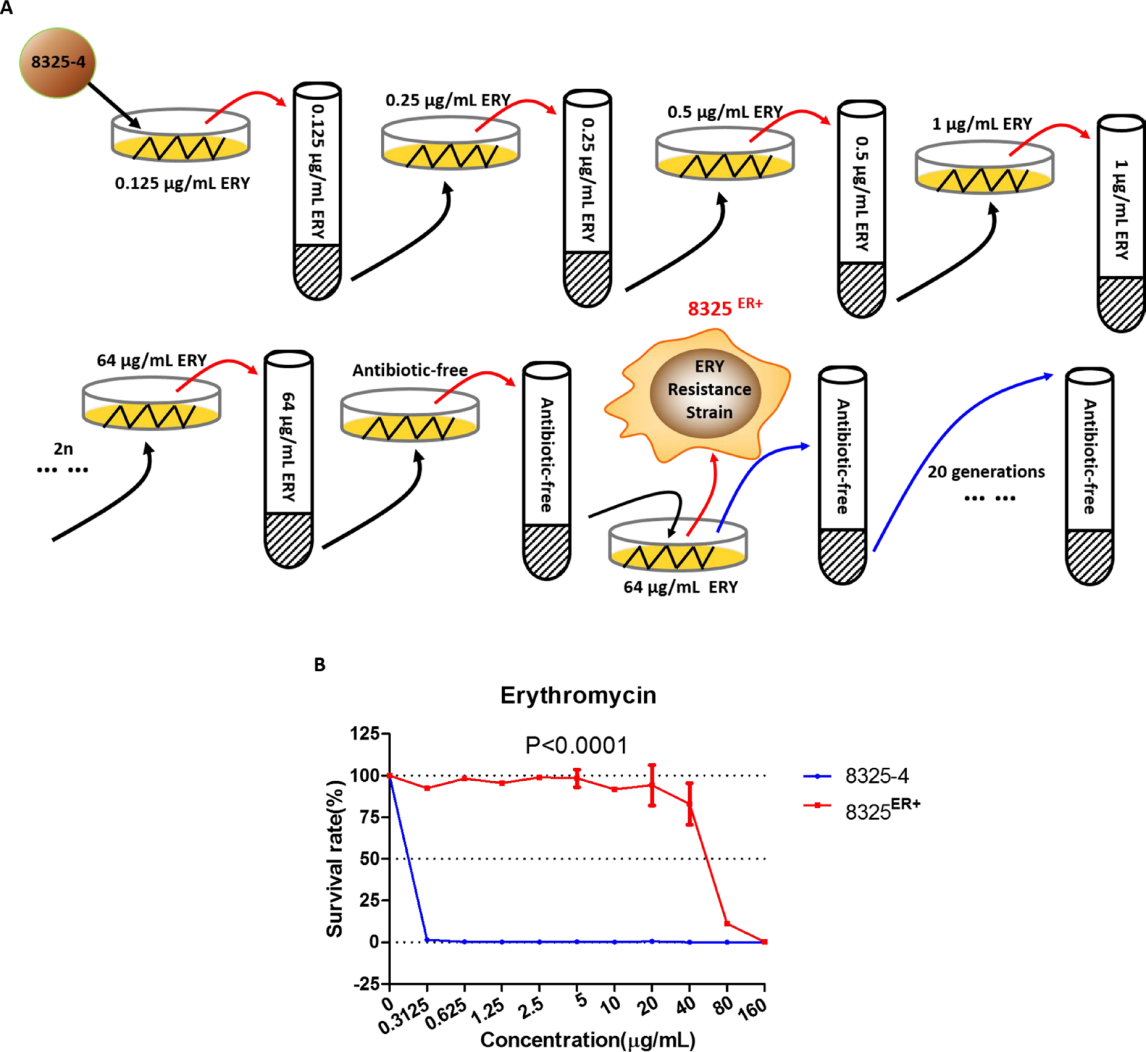
Erythromycin-resistant strain 8325^ER+^ (**A**) Schematic diagram of stepwise screening for resistance to erythromycin (ERY) of wild-type *S. aureus*. 8325–4 was cultured and passaged in BHI medium supplemented with various concentrations of erythromycin (initially 0.125 μg/mL, followed by two-fold increased until the concentration of erythromycin reached 64 μg/mL). Each screening step included solid and liquid BHI medium for screening. Solid medium was used for picking an isolate of *S. aureus* at 37° C incubator for 12 h, and liquid medium was used for enrichment of the isolate at 37° C with shaking at 220 rpm. When screening was completed, the erythromycin-resistant isolate was inoculated in BHI medium without antibiotics for 20 generations. (**B**) Survival rates of 8325–4 and 8325^ER+^ in different concentration of erythromycin. The survival curve of wild-type 8325–4 is shown in blue and the 8325^ER+^ strain in red. Values are the means of triplicate wells; error bars indicate SD.

To explore the mechanism underlying the resistance to macrolide in *S. aureus*, we cultured wild-type *S. aureus* 8325-4 in BHI medium while continuously doubling the concentration of erythromycin (Figure 1A). An isolate with acquired resistance to erythromycin was screened and named 8325^ER+^. It was able to grow in a medium containing 80 μg/mL of erythromycin, and the survival rates of 8325^ER+^ strain in different concentration of erythromycin were significantly higher than the parent strain (Figure 1B). Besides, the minimal inhibitory concentration (MIC) of erythromycin of 8325^ER+^ was 160 μg/mL in a drug susceptibility test, which was interpreted as erythromycin resistant according to Clinical and Laboratory Standards Institute (CLSI) criteria [24].

In the further investigation, 8325^ER+^ was cultured in BHI broth without erythromycin for 20 generations, the susceptibility to erythromycin of bacteria from different generations was determined individually. We found that the MIC of *S. aureus* from different generations was not altered, which suggested that resistance to erythromycin in 8325^ER+^ was inheritable.

### Whole-genome sequencing identifies gene mutations in 8325^ER+^

To test whether high expression of known erythromycin resistance genes in 8325^ER+^ contributed to the resistance to erythromycin, we extracted total RNA from the erythromycin-sensitive strain 8325-4 and the erythromycin-resistant strain 8325^ER+^ (Supplementary Figure 1A). Reverse transcription-polymerase chain reaction (RT-PCR) showed that erythromycin resistance-related genes, including 23 S rRNA adenine-specific *N*-methyltransferases (encoded by *ermA*/*ermB*/*ermC*), *mphC*, and *msrA* were not detected in 8325^ER+^ (Supplementary Figure 1B). These results suggest that another mechanism is responsible for the resistance to erythromycin of 8325^ER+^.

To investigate the potential genes involved in the resistance occurrence to erythromycin, we extracted total genomic DNA and compared the genome sequence of 8325^ER+^ with that of 8325-4.

Sequence analysis showed that ten genes mutated, and six of them encoded different proteins (Table 1). Considering that five of the ten mutated genes were involved in the translation process, we decided to compare cell growth between 8325^ER+^ and 8325-4. We did not see a significant difference between both strains (Supplementary Figure 1C).

**Table 1:** Comparison of whole-genome sequence of 8325^ER+^ with 8325–4

Type of mutation	Nucleotide	Amino acid	Locus tag	Product	Position in chromosome
Point mutation	T to A	—	—	Noncoding	75324 of CP000253.1
	T 102 G	G 34 G	SAOUHSC_01078	ribosomal protein L32	1042000 of CP000253.1
	C 1988 T	S 663 L	SAOUHSC_01583	conserved hypothetical phage protein	1508580 of CP000253.1
	C 530 G	A 177 G	SAOUHSC_01748	queuine tRNA-ribosyltransferase	1653225 of CP000253.1
	A 184 G	R 62 G	SAOUHSC_02163	conserved hypothetical phage protein	2031924 of CP000253.1
	G to A	—	SAOUHSC_R0005	16S ribosomal RNA	2243146 of CP000253.1
	A 208 C	T 70 P	SAOUHSC_02511	ribosomal protein L4	2316907 of CP000253.1
	G 206 C	G 69 A	SAOUHSC_02511	ribosomal protein L4	2316909 of CP000253.1
	A to G	—	—	Noncoding	2350008 of CP000253.1
Fragment insertion	C 291 or 318 to CAAACGTACAAGCCA CATTACAATCGTC	KRTSHITIV	SAOUHSC_02507	ribosomal protein L22	2314658 of CP000253.1

**Figure 2:**
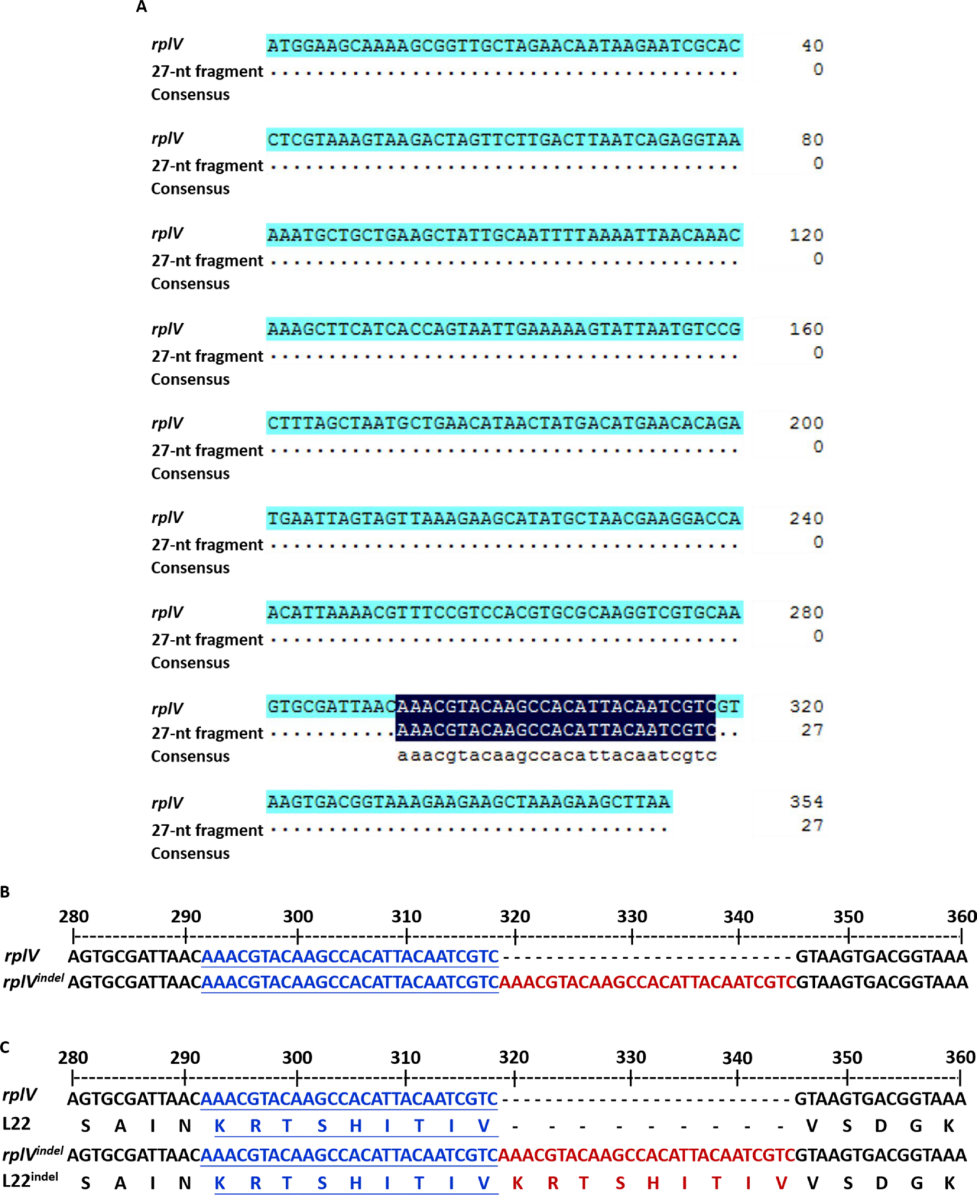
Insertion of a 27 nucleotide-repeat fragment in the *rplV* gene (**A**) BLAST analysis of 27-nt fragment sequence. The top line shows the *rplV* gene, and the middle line shows 27-nt fragment sequences. BLAST results are shown for the total *rplV* nucleotides. (**B**) Nucleotides alignment of partial sequences of wild-type *rplV* and *rplV^indel^* to display the region 27-nt fragment insertion. The letters in blue and underlined on the middle line and bottom line are coincide with the inserted 27-nt fragment shown in red on the bottom line, and the 27-nt fragment is exactly adjacent to the region 292–318 of *rplV*. (**C**) Protein alignment of partial sequences of wild-type L22 and L22^indel^ to display the inserted 27-nt fragment that led to a 9-amino acid insertion without frame-shifting mutation. The letters in blue and underlined are coincide with the inserted fragment shown in red on the line.

Further analysis showed that seven genes were identified with one or two nucleotide mutations, and only one gene, *rplV*, encoding ribosomal protein L22 exhibited an insertion of a 27-nt fragment (Table 1). In further investigation, we found that sequence of the insertion segment correspond to a duplication of the region 292–318 of *rplV* (Figure 2A). Moreover, the insertion of the 27-nt fragment happen at nucleotide 292C or 318C in *rplV* gene in 8325^ER+^ (Figure 2B). Furthermore, we found that the 27-nt fragment led to a 9-amino acids insertion but did not induce a frame-shifting mutation (Figure 2C).

### Twenty-seven-nucleotide insertion in the *rplV* (*rplV^indel^*) gene induces resistance to erythromycin in *S. aureus*

**Figure 3:**
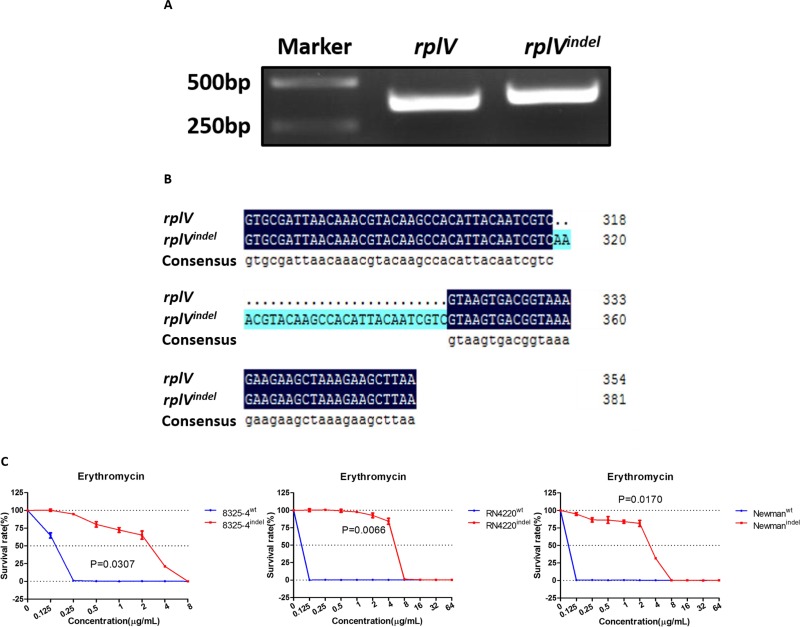
*rplV^indel^* gene contributes to resistance to erythromycin in drug-susceptible *S. aureus* (**A**) *rplV* genes were amplified by PCR from the genomes of 8325–4 and 8325^ER+^, and PCR products were resolved on a 2% agarose gel and visualized by ultraviolet imaging. (**B**) The nucleotides were sequenced by Sangon Biotech, and BLAST analysis was performed using DNAMAN. The top line shows wild-type *rplV*, and the middle line shows *rplV^indel^*. BLAST results are shown for part of the total *rplV* nucleotides. (**C**) Survival rates of recombinant 8325–4, RN4220, and Newman cells in different concentration of erythromycin. Drug-susceptible *S. aureus* cells transformed with the *rplV^indel^* gene exhibit decreased sensitivity to erythromycin. The survival curve of cells harboring wild-type *rplV* gene is shown in blue and the *rplV^indel^* gene is shown in red. Values are the means of triplicate wells; error bars indicate SD.

The *rplV* gene encodes the ribosomal 50S subunit protein L22, which is important for ribosomal 50S subunit assembly at the early stage. It is essential for the formation of the nascent peptide exit tunnel of the mature ribosome [22]. Given that mutation in the *rplV* gene was reported to be involved in resistance to antibiotics in *E. coli* and *S. aureus* [11, 21–23], we focused on investigating whether the *rplV^indel^* gene induced resistance to erythromycin. Firstly, the *rplV* genes were amplified by PCR from the genomes of 8325^ER+^ and 8325-4. We found the band of PCR product from 8325^ER+^ was bigger than that from 8325-4 (Figure 3A). Following analysis showed that the sequence of *rplV* gene in 8325^ER+^ containing the 27-nt insertion fragment, which was consistent with the genome sequence results (Figure 3B).

To evaluate the role of *rplV^indel^* in raising resistance of *S. aureus* to erythromycin, we generated several erythromycin-susceptible *S. aureus* strains (8325-4^indel^, RN4220^indel^, and Newman^indel^) with ectopic expression of *rplV^indel^*. Meanwhile, these strains transferred with the wild-type *rplV* gene were used as control (Supplementary Figure 2). We found that the survival rates of *S. aureus* strains with ectopic expression of *rplV^indel^* in different concentration of erythromycin were significantly higher than control strains (Figure 3C). And erythromycin MICs in 8325-4^indel^, RN4220^indel^, and Newman^indel^ were 8 μg/mL respectively (Table 2), which suggests that ectopic expression of *rplV^indel^* in susceptible strains induced resistance to erythromycin.

**Table 2:** Antimicrobial agent susceptibility of *Staphylococcus* strains

Antimicrobial Agents	MIC (μg/mL)^*^							
	8325–4				RN4220		Newman	
	8325–4	8325^ER+^	8325–4^wt^	8325–4^indel^	RN4220^wt^	RN4220^indel^	Newman^wt^	Newman^indel^
Erythromycin	0.3125^S^	160^R^	0.25^S^	8^R^	0.125^S^	8^R^	0.125^S^	8^R^
Azithromycin	0.78125^S^	200^R^	0.5^S^	16^R^	0.5^S^	32^R^	0.5^S^	32^R^
Clarithromycin	0.1953125^S^	100^R^	0.125^S^	8^R^	0.125^S^	8^R^	0.125^S^	8^R^
Chloramphenicol	5	5	−	−	−	−	−	−
Linezolid	1.25	1.25	0.625	0.625	1	1	1	1
Tobramycin	1.25	1.25	2.5	2.5	2	2	2	2
Kanamycin	5	5	5	5	8	8	8	8
Vancomycin	1.25	1.25	1	1	1	1	2	2

^*^The MICs of corresponding antibiotic shown with letter “R” represent “Resistant”, those with letter “S” represent “Susceptible” according to CLSI criteria.

### *rplV^indel^* contributes to specific resistance to macrolides in *S. aureus*

**Figure 4:**
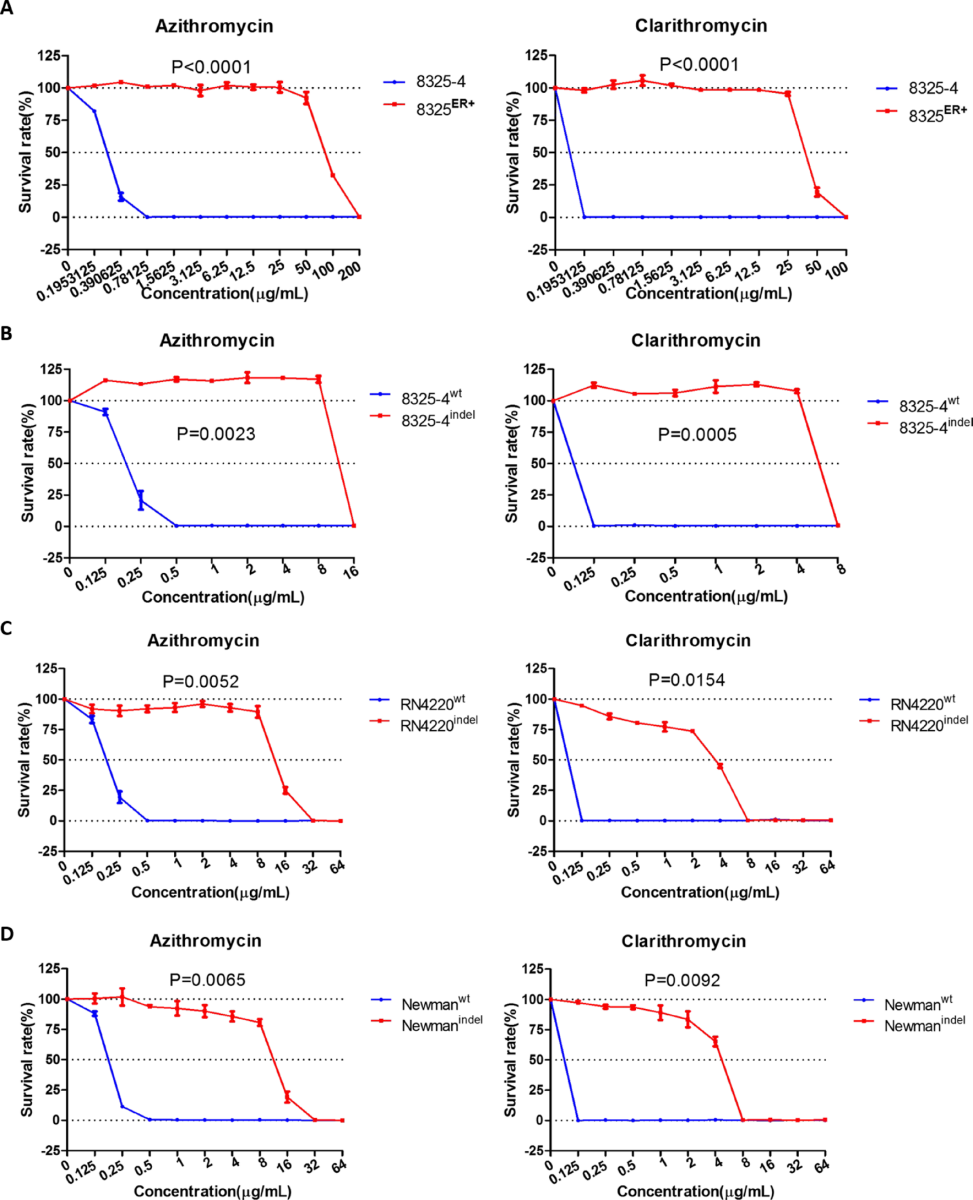
*rplV^indel^* gene contributes to resistance to macrolides in drug-susceptible *S. aureus* (**A**) Survival rates of 8325 and 8325^ER+^ in different concentration of azithromycin (left) and clarithromycin (right). (**B**) Survival rates of wild-type *S. aureus* 8325–4 cell transformed with the *rplV^indel^* gene exhibit decreased sensitivity to azithromycin (left) and clarithromycin (right). (**C**) Survival rates of drug-susceptible RN4220 cell transformed with the *rplV^indel^* gene exhibit decreased sensitivity to azithromycin (left) and clarithromycin (right). (**D**) Survival rates of drug-susceptible Newman cell transformed with the *rplV^indel^* gene exhibit decreased sensitivity to azithromycin (left) and clarithromycin (right). The survival curve of cells harboring wild-type *rplV* gene is shown in blue and the *rplV^indel^* gene is shown in red. Values are the means of triplicate wells; error bars indicate SD.

Next, we asked whether the *rplV^indel^* gene could induce resistance to other macrolides, including azithromycin and clarithromycin. First, we also found the same results with erythromycin susceptibility test. The survival rates of 8325^ER+^ strain in different concentration of azithromycin and clarithromycin were significantly higher than parent strain (Figure 4A). We then determined the MIC of azithromycin and clarithromycin in 8325^ER+^ as 200 μg/mL and 100 μg/mL, respectively (Table 2). In line with expectation, the ectopic expression of *rplV^indel^* in susceptible strains also induced resistance to these two antibiotics (Figure 4B–4D, Table 2).

As the ribosomal protein L22 is essential in formation of the ribosomal polypeptide exit tunnel [22], we then determined whether *rplV^indel^* was involved in resistance to antibiotics targeting the ribosome. It was revealed that neither 8325^ER+^ nor susceptible strains with ectopic expression of *rplV^indel^* were resistant to chloramphenicol and linezolid (Table 2), which target the 50 S ribosomal subunit. Similar results were also obtained in a drug susceptibility test of antibiotics that target the 30 S subunit or cell wall (Table 2). Consistent with the MICs of non-macrolide antibiotics of 8325^ER+^ strain or susceptible strains with ectopic expression of *rplV^indel^*, there were no significant differences observed among the survival rates of those *S. aureus* strains comparing with their control strains (Supplementary Figures 3A–3B, 4A–4C).

## DISCUSSION

Macrolides are usually used in clinical therapy for skin infections caused by *S. aureus*. Several mechanisms involved in *S. aureus* resistance to macrolides have been revealed. In this study, we revealed that a 27-nt insertion in the *rplV* gene induced a specific resistance to macrolides.

The 8325^ER+^ resistance to macrolides was not due to the occurrence of identified erythromycin-resistant genes (*ermA*/*ermB*/*ermC*/*mphC*/*msrA*) but the *rplV^indel^* gene. Interestingly, the 27-nt insertion sequence is a repeat sequence of the *rplV* gene, but it did not induce frame-shifting mutation. Sequence analysis showed that this fragment might be inserted behind 291C or 318C. The ectopic expression of the *rplV^indel^* gene in several susceptible strains specifically conferred resistance to macrolide antibiotics. As shown in Table 2, the MIC of macrolides was higher in 8325^ER+^ than in 8325^indel^. We think this may be majorly due to the coexistence of *rplV* and *rplV^indel^*. The ribosome consisting of ribosomal protein L22 (*rplV*) is still sensitive to macrolides. Mutations in other genes may also contribute the resistance to macrolides in 8325^ER+^. These assumptions will be investigated in the future.

In 1967, bacterial resistance to macrolides, caused by mutations in ribosomal protein, was reported [25]. In *E. coli*, it has been found that the deletion of M^82^K^83^R^84^ increases expression of the AcrAB-TolC efflux system and results in resistance to macrolides [21, 26]. Here, we reveal that a 27-nt insertion in the *rplV* gene confers specific resistance to macrolides in *S. aureus*. However, the level of *msrA*, a well-identified gene of the efflux system involved in resistance to macrolides in staphylococci [17, 18], was not altered in 8325^ER+^ compared with that of 8325-4. The resistance to macrolides induced by *rplV^indel^* may be due to the conformational changes of L22 protein induced by the 27-nt insertion, which will be investigated in the future.

Ribosome protein L4 forms part of the lining of the peptide exit tunnel with L22. Mutations in ribosome protein L4 also induce macrolides resistance in a variety of pathogenic and non-pathogenic bacteria [21, 27–29]. There are two amino acids mutations (G69A, T70P) identified in the L4 protein from 8325^ER+^, which may also contribute to the macrolides resistance. Although the ectopic expression of the *rplV^indel^* gene in several susceptible strains specifically conferred resistance to macrolide antibiotics, combination with the mutant L4 protein may further elevate the resistance.

In this study, we tried to detect *rplV^indel^* in 84 clinical *S. aureus* isolates resistant to macrolides. Most of these strains harbor *ermA/B/C* genes (Supplementary Table 1). There was no strain identified that contained the *rplV^indel^* genes (data not shown). These results indicate that the occurrence of *rplV^indel^* in clinical isolates is rare compared with that of *erm* genes.

In conclusion, our findings present a 27-nt insertion in *rplV* that induces the specific resistance to macrolides in *S. aureus.*

## MATERIALS AND METHODS

### Bacterial strains, plasmids, and growth conditions

The strains and plasmids used in this study are listed in Supplementary Table 2. 84 Clinical macrolides-resistant *S. aureus* obtained from Department of Clinical Laboratory, Peking University People's Hospital. Distributions of the 84 clinical samples of *S. aureus* by origin of recovery were 22 strains from blood (26.2%), 11 strains from pus (13.1%), 14 strains from secretions (16.7%), 27 strains from sputum (32.1%), 9 strains from wound (10.7%) and one strain from abdominal fluid (1.2%). Strains were cultured using brain heart infusion (BHI) medium (BD) at 37°C for 12 h with shaking at 220 rpm. Clinical isolates and wild-type strains including 8325-4, RN4220 and Newman were cultured in antibiotic-free BHI broth, while 8325^ER+^ strain was cultured in BHI broth supplemented with 50 μg/mL erythromycin, those wild-type strains transformed with the shuttle plasmid pOS1 supplemented with 25 μg/mL chloramphenicol, *E. coli* strain transformed with a cloning plasmid pMD-19T supplemented with 100 μg/mL ampicillin.

### Erythromycin screen *in vitro*

Schematic diagram of stepwise screening for resistance to erythromycin of wild-type *S. aureus* was showed in Figure 1A. 8325-4 was cultured and passaged in BHI medium supplemented with various concentrations of erythromycin (initially 0.125 μg/mL, followed by two-fold increased until the concentration of erythromycin reached 64 μg/mL). Each screening step included solid and liquid BHI medium for screening. Solid medium was used for picking an isolate of *S. aureus* at 37°C incubator for 12 h, and liquid medium was used for enrichment of the isolate at 37°C with shaking at 220 rpm. When screening was completed, the erythromycin-resistant isolate was inoculated in BHI medium without antibiotics for 20 generations.

### Measurement of bacterial growth curve

Bacteria were incubated in BHI broth at 37°C with shaking at 220 rpm overnight. The concentration of bacteria was adjusted to 1 × 10^7^ cfu/mL, then 1:100 inoculated in BHI broth without antibiotics at 37°C with shaking at 220 rpm for 12 hours. Growth curves of bacteria were constructed by measuring of the cell density at A600 nm at one-hour intervals for 12 hours.

### Antibiotic susceptibility assay

Antibiotics were purchased from Selleck. Susceptibility to antibiotics was tested by using broth microdilution method according to the Clinical and Laboratory Standards Institute (CLSI) [24]. Briefly, antibiotics were prepared by serial two-fold dilutions in BHI broth, then various concentration of antibiotics were made in triplicate in 96-well culture dishes containing 1 × 10^5^ cfu/well bacteria and incubated for 18–24 h at 37°C. Control wells were free of antibiotic. Bacteria growth was determined by reading the optical density (OD) at 630 nm. The survival rates of bacteria were calculated by the rates of OD 630 nm measurement at each concentration of antibiotic versus control wells. The MIC was determined to be the dose of antibiotic that inhibited bacteria growth by >95%.

### RNA isolation and RT-PCR

For detecting erythromycin resistance genes in *S. aureus*. Total bacterial RNA was extracted from *S. aureus*, which were grown with shaking at 37°C using Trizol (Invitrogen) as previously described [30]. Briefly, DNase digestion of 80 μL of total RNA was performed with 10U of RNase-free DNase I (Promega) and 10 μL of the 10 × reaction buffers in a total reaction volume of 100 μL for 30 min at 37°C. For cDNA synthesis, 6 μL total RNA (≈250 ng) was incubated at 65°C for 5 min, then add 2 μL of 4 × DNA remove buffer and incubate at 37°C for 5 min, finally add 2 μL of 5 × RT Master MixII (TOYOBO) and incubate at 37°C for 15 min, 50°C for 5 min, 98°C for 5 min.

### Detection of macrolides-resistance genes

Macrolides resistance genes *ermA*, *ermB*, *ermC*, *msrA* and *mphC* were examined in the erythromycin-sensitive strain 8325-4 and the erythromycin-resistant strain 8325^ER+^ with primers listed in Supplementary Table 3. The PCR reaction mixture contained 2.5 μL of 10 × PCR reaction buffer, 0.25 μL enzyme, 0.5 μL dNTP mix, 0.3 mM of gene-specific forward and reverse primers, and 2 μL of template, made up to a final volume of 25 μL with distilled water. Cycling parameters were set as follows: initial activation step at 95°C for 5 min, denaturation at 95°C for 30 s, annealing at 55°C for 30 s, and extension at 72°C for 30 s. *gyrB* was used as the endogenous reference gene. The PCR products were resolved in 2% agarose gel and visualized by ultraviolet imaging.

### Whole-genome sequencing of *S. aureus*

Bacteria were grown in BHI broth at 37°C for 12 h with shaking at 220 rpm and harvested by centrifuge at 12000 rpm for 1 min. Genomic DNA was extracted by using EasyPure^®^ Bacteria Genomic DNA Kit (TransGen Biotech) according to manufacturer's instruction. Sequencing with constructed shotgun libraries of 8325-4 and 8325^ER+^ was performed by Illumina Hiseq 2000. Fragmentation, library construction, and sequencing were carried out by oebiotech company.

### Analysis of the *rplV* gene in clinical isolates

For detecting 27-nt fragment insertion in *rplV* gene in clinical macrolides resistant *S. aureus* isolates, the *rplV^indel^* gene was detected by PCR amplification. Primers used for the *rplV* gene were *rplV*-F and *rplV*-R listed in Supplementary Table 3. Clinical isolates were grown on blood agar plates and incubated overnight at 37°C, bacteria DNA was prepared by suspending a fresh colony in 400 μL of sterile distilled water and heating at 100°C for 10 min and then centrifuged at 12000 rpm for 5 min. PCR amplification was carried out under the following conditions: 95°C for 5 min, followed by 34 cycles of 95°C for 30 s, 56°C for 30 s, 72°C for 30 s, and 72°C for 5 min. PCR products were resolved on a 2% agarose gel and visualized by ultraviolet imaging. The nucleotides were sequenced by Sangon Biotech, and BLAST analysis was performed using DNAMAN.

### Ectopic expression of *rplV^indel^* in *S. aureus* strains

*rplV* genes were amplified by PCR from the genomes of wild-type *S. aureus* 8325-4 and the erythromycin-resistant strain 8325^ER+^ with primers *rplV*-F-*EcoR*I and *rplV*-R-*BamH*I (Supplementary Table 3). The PCR products were ligated into pMD-19T vector, the recombinant plasmids were transformed into DH5α. The recombinant pMD-19T plasmid was eliminated by cutting the plasmid with the *EcoR*I and *BamH*I restriction enzymes, then digested fragments were ligated into *EcoR*I and *BamH*I-digested pOS1 vector. The recombinant plasmids were transformed into DH5α, then electro-transformation into recipient strains *S. aureus* RN4220. The plasmid was isolated from RN4220, then electro-transformation into *S. aureus* 8325-4 and Newman.

### Statistical analyses

Statistical tests were performed using GraphPad Prism v.5.0 (GraphPad Software Inc., San Diego, CA, United States). The Differences between survival curves were evaluated for statistical significance using the unpaired *t* test. All *P*-values of ≤ 0.05 was considered significant.

## SUPPLEMENTARY MATERIALS TABLES AND FIGURES



## Abbreviations

*rplV*: ribosomal protein L22
*rplV^indel^*: 27-nucleotide repeat sequence insertion in *rplV* gene
rRNA: ribosomal RNA
*erm*: erythromycin resistance methyltransferase
ABC: ATP-binding cassette
*msrA*: macrolide-streptogramins resistance A
*mphC*: macrolide phosphotransferase C
RT-PCR: reverse transcription-polymerase chain reaction
ERY: erythromycin
MIC: minimal inhibitory concentration
CLSI: Clinical and Laboratory Standards Institute.
