# Supplementary material for: Twenty-seven-nucleotide repeat insertion in the rplV gene confers specific resistance to macrolide antibiotics in Staphylococcus aureus
Source: Oncotarget. 2018 May 25;9(40):26086–95. doi: 10.18632/oncotarget.25441 (PMC5995244; doi:10.18632/oncotarget.25441)
Supplement: Supplementary file 1 [file oncotarget-09-26086-s001.pdf]

Twenty-seven-nucleotide repeat insertion in the *rplV* gene confers specific resistance to macrolide antibiotics in *Staphylococcus* aureus

SUPPLEMENTARY MATERIALS

Supplementary Table 1: Detection of erythromycin resistance genes in clinical *S.aureus* isolates (n = 84)

erythromycin resistance genes			No. of isolates (%)
ermA	***ermB***	***ermC***	
+	+	+	1 (1.2)
+	+	−	0 (0)
+	−	+	13 (15.5)
+	−	−	8 (9.5)
−	+	+	5 (6.0)
−	+	−	4 (4.8)
−	−	+	36 (42.8)
−	−	−	17 (20.2)

Supplementary Table 2: Strains and plasmids used in this study

Strain or plasmid	Comments	Source or reference
Strains		
S. aureus		
8325-4	Wild-type	[1]
RN4220	Restriction-negative strain, 8325 derivative	[2]
Newman	Clinical isolate	[3]
8325^{ER+}	Wild-type 8325 resistance to macrolides	This study
$8325\text{-}4^{wt}$	8325-4 harboring pOS1-*rplV*	This study
$8325\text{-}4^{indel}$	8325-4 harboring pOS1-$rplV^{indel}$	This study
$RN4220^{wt}$	RN4220 harboring pOS1-*rplV*	This study
$RN4220^{indel}$	RN4220 harboring pOS1-$rplV^{indel}$	This study
$Newman^{wt}$	Newman harboring pOS1-*rplV*	This study
$Newman^{indel}$	Newman harboring pOS1-$rplV^{indel}$	This study
E. coli		
DH5α	A host strain for cloning	Transgene
Plasmids		
pMD-19T	*E. coli* cloning vector, Amp^R	TaKaRa
pOS1	*E. coli* - *S. aureus* shuttle vector, Cm^R	[4]
pOS1-*rplV*	pOS1 with the *rplV* coding sequence from 8325-4, Cm^R	This study
pOS1-$rplV^{indel}$	pOS1 with the $rplV^{indel}$ coding sequence from 8325^{ER+}, Cm^R	This study

Abbreviations: wt, wild-type; indel, 27-nucleotide repeat insertion; Amp^R, ampicillin-resistant; Cm^R, chloramphenicol-resistant.

Supplementary Table 3: Primers used in this study

Primer/sequence	Oligonucleotide sequence (5′-3′)	Source or reference
rplV-F	ATGGAAGCAAAAGCGGTT	This study
rplV-R	TTAAGCTTCTTTAGCTTC	
rplV-F-*EcoR*I	GATATGCATGAATTCATGGAAGCAAAAGCG	This study
rplV-R-*BamH*I	GGATCCTTAAGCTTCTTTAGC	
gyrB RT-F	TTATGGTGCTGGGCAAATACA	[5]
gyrB RT-R	CACCATGTAAACCACCAGATA	
ermA RT-F	CAAAGCCTGTCGG	[6]
ermA RT-R	TCATCCTAAGCCAAGT	
ermB RT-F	TTACTTTGGCGTGTTT	[7]
ermB RT-R	AGGGTTGCTCTTGC	
ermC RT-F	AACCCATTTCATAACG	[8]
ermC RT-R	AATACAAAACGCTCAT	
msrA RT-F	ATTCGCTTTCGTTG	[9]
msrA RT-R	ATGGCATACTATCGTCA	
mphC RT-F	CGCCGATTCTCCT	[10]
mphC RT-R	ACTGAAGCAACCCAC	

Abbreviations: F, forward; R, reverse; RT: reverse transcription.

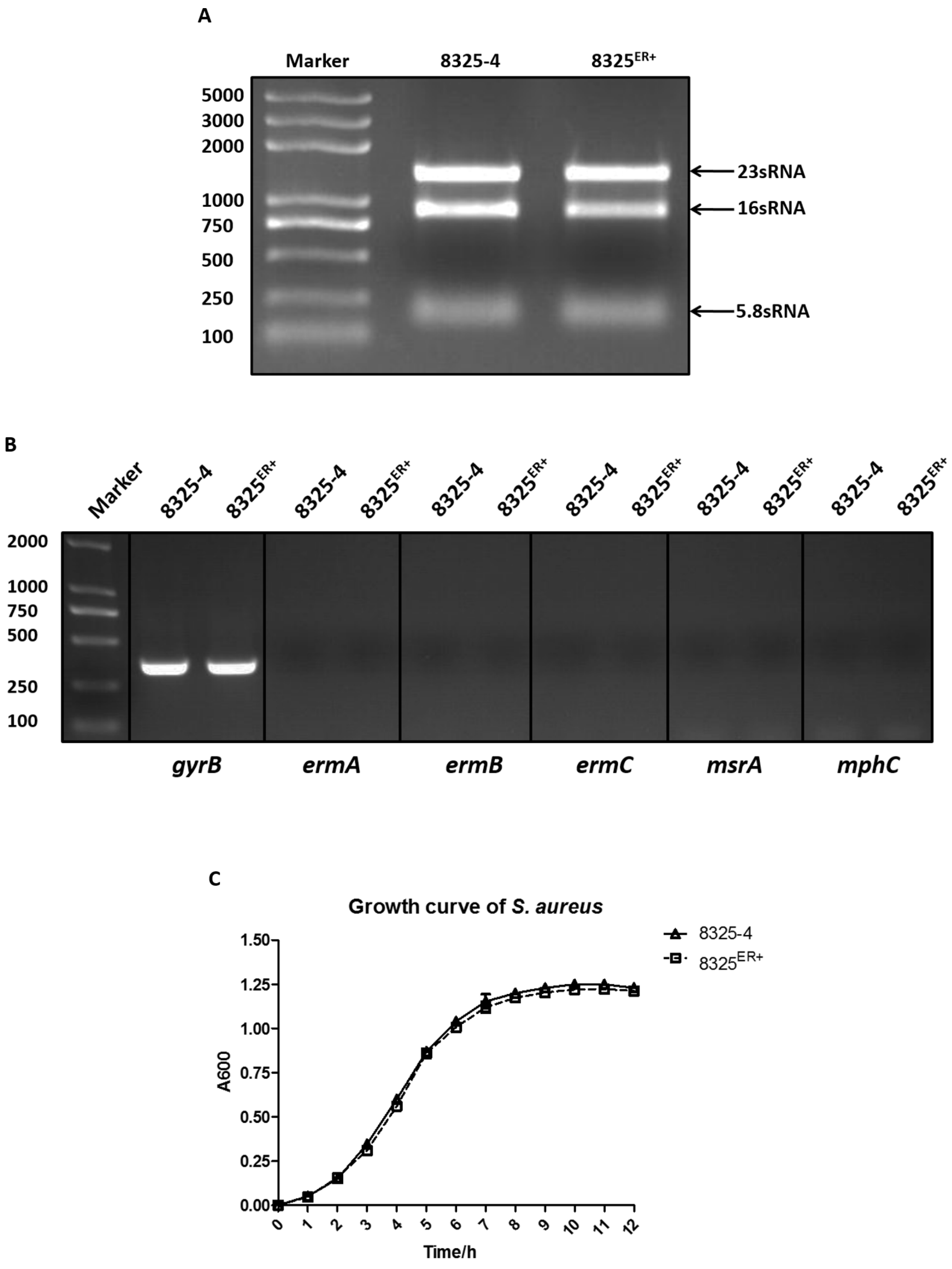

Supplementary Figure 1: Detection of erythromycin-resistant genes in strain 8325^{ER+}. (**A**) Total RNA extraction from the 8325-4 and 8325^{ER+} strains cultured for 12 h was carried out according to protocols described in methods. (**B**) Erythromycin resistance genes were tested by RT-PCR according to protocols described in methods. All the genes are marked below the corresponding line. *gyrB* was used as the endogenous reference gene. The RT-PCR products were resolved on a 2% agarose gel and visualized by ultraviolet imaging. (**C**) Growth curves of erythromycin-susceptible strain (8325-4) and erythromycin-resistant strain (8325^{ER+}). Bacteria were cultured in BHI medium without erythromycin at 37° C with shaking at 220 rpm, and the cell density was determined by measurements at 600 nm every hour for 12 h. Error bars represent the means of three independent experiments.

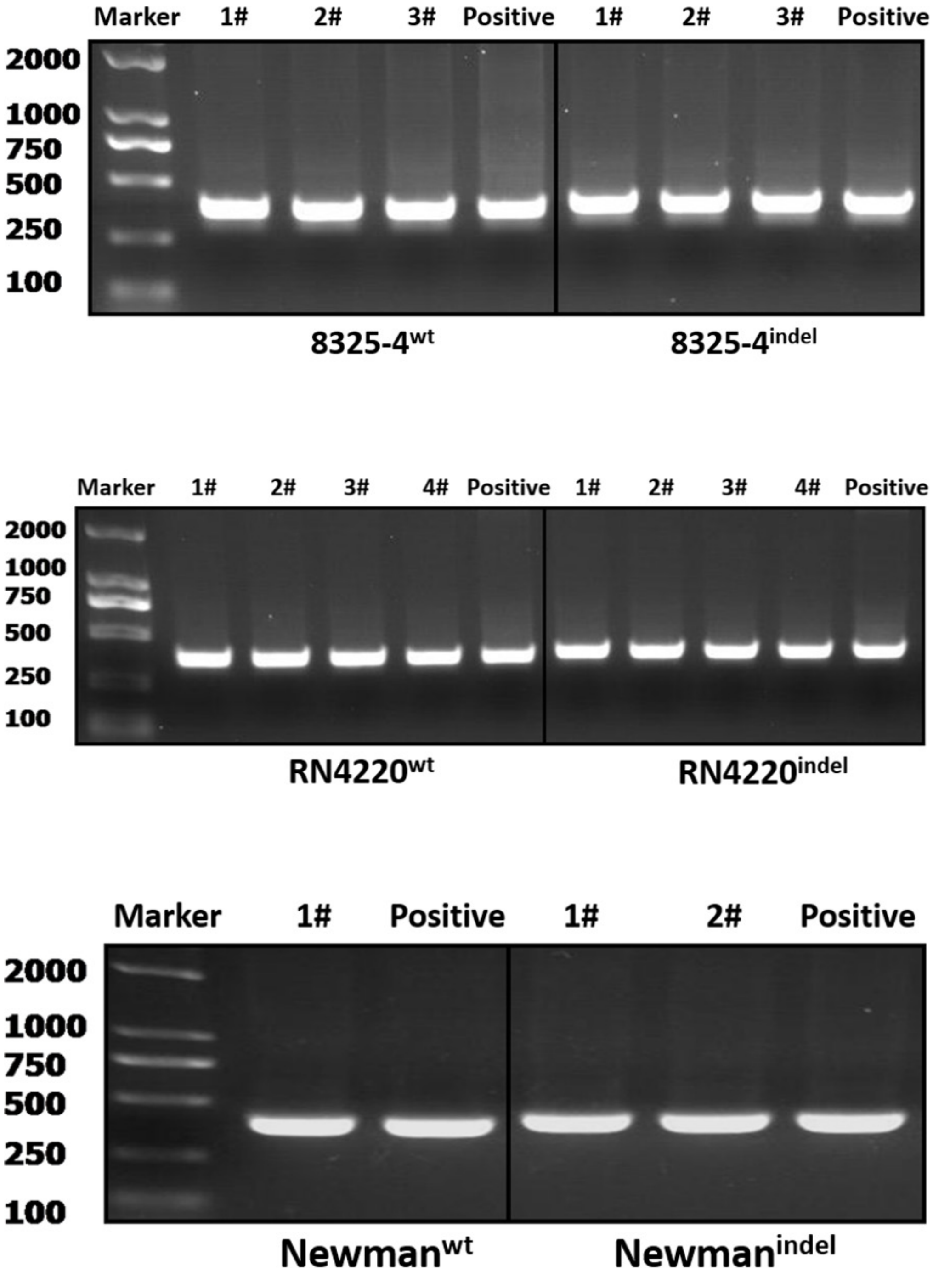

Supplementary Figure 2: Erythromycin-susceptible *S. aureus* cells transformed with the *rplV*^{indel} gene. PCR was used to assess the presence of the *rplV* or *rplV*^{indel} gene in 8325-4, RN4220, and Newman cells transformed with the pOS1-*rplV* or pOS1-*rplV*^{indel} vector. Genomes of the 8325-4 or 8325^{ER+} strain were used as controls. Plasmids of different isolates grown in the chloramphenicol BHI agar plate were used as templates marking # at the top. The PCR products were resolved on a 2% agarose gel and visualized by ultraviolet imaging.

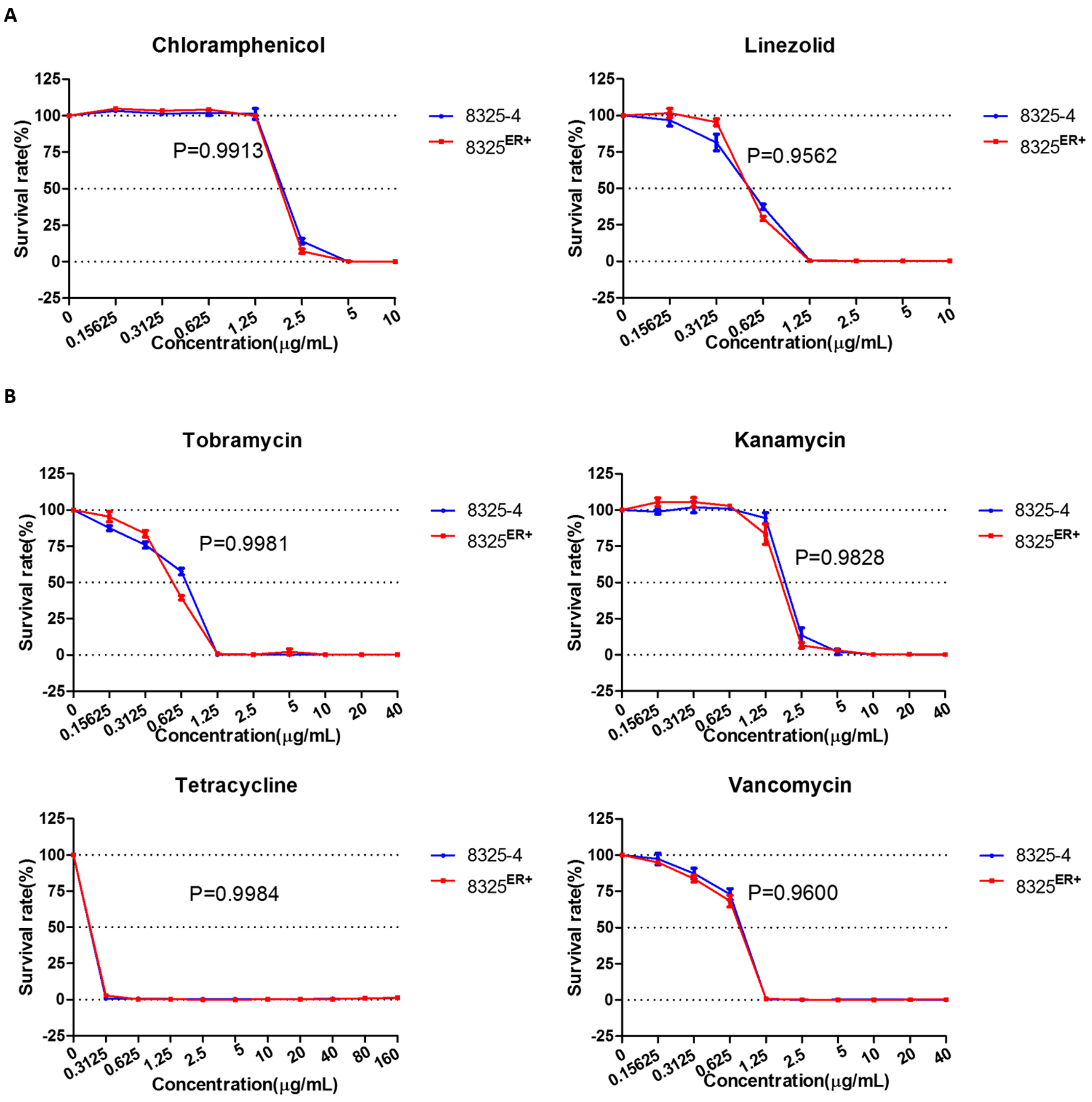

Supplementary Figure 3: 8325^{ER+} is susceptible to non-macrolide antibiotics. (**A**) Survival rates of 8325 and 8325^{ER+} in different concentration of chloramphenicol (left) and linezolid (right). (**B**) Survival rates of 8325 and 8325^{ER+} in different concentration of tobramycin, kanamycin, tetracycline, and vancomycin. The survival curve of wild-type 8325-4 is shown in blue and the 8325^{ER+} strain is shown in red. Values are the means of triplicate wells; error bars indicate SD.

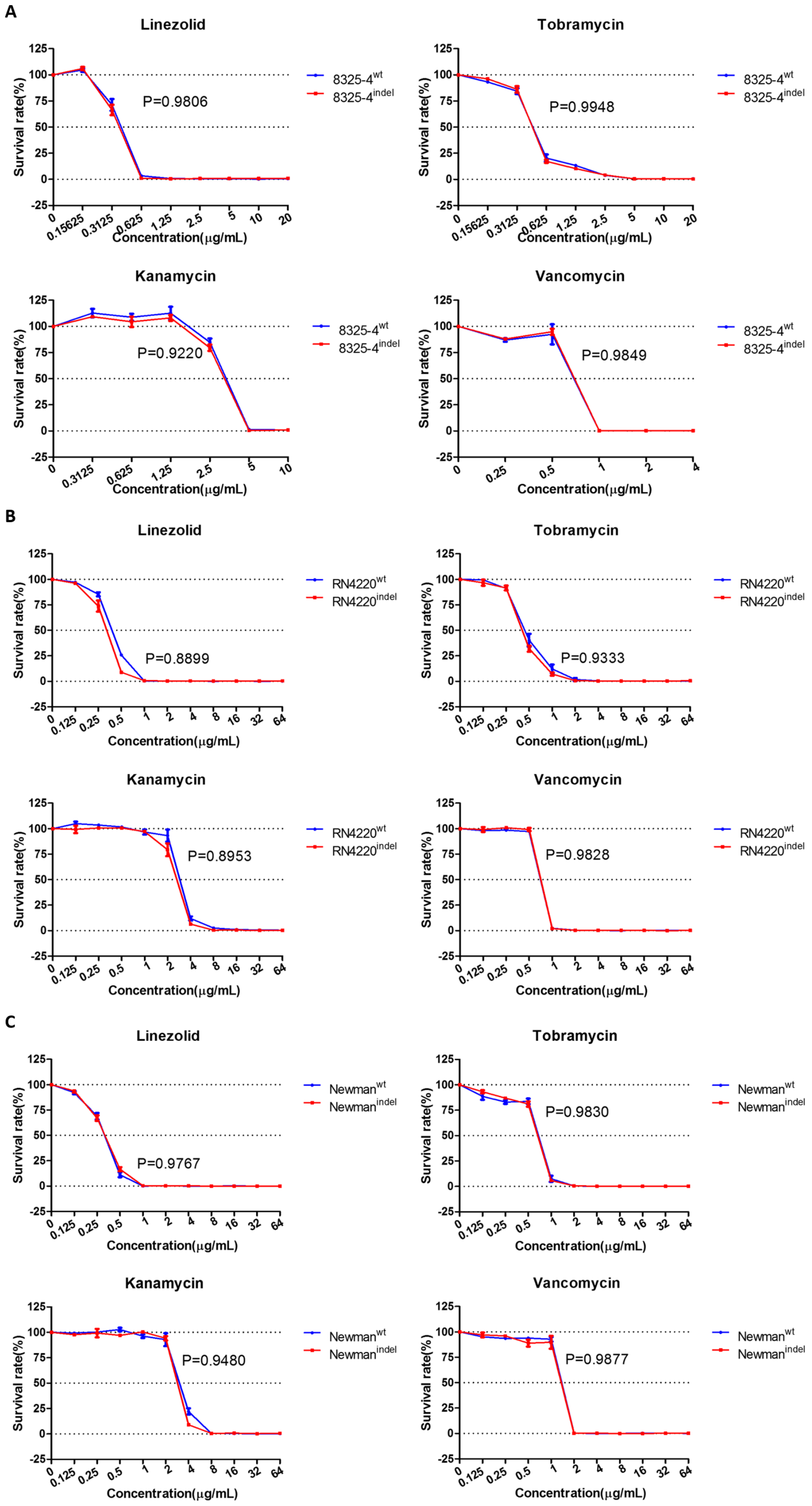

Supplementary Figure 4: *rplV*^{indel} gene does not change *S. aureus* susceptibility to non-macrolide antibiotics. (**A**) Survival rates of wild-type *S. aureus* 8325-4 transformed with the *rplV*^{indel} gene exhibit no change in its susceptibility to non-macrolide antibiotics. (**B**) Survival rates of RN4220 cell transformed with the *rplV*^{indel} gene exhibit no change in susceptibility to non-macrolide antibiotics. (**C**) Survival rates of Newman cell transformed with the *rplV*^{indel} gene exhibit no change in susceptibility to non-macrolide antibiotics. The survival curve of cells harboring wild-type *rplV* gene is shown in blue and the *rplV*^{indel} gene is shown in red. Values are the means of triplicate wells; error bars indicate SD.

REFERENCES

1. Novick R. Properties of a cryptic high-frequency transducing phage in Staphylococcus aureus. Virology. 1967; 33:155–166.
2. Kreiswirth BN, Lofdahl S, Betley MJ, O'Reilly M, Schlievert PM, Bergdoll MS, Novick RP. The toxic shock syndrome exotoxin structural gene is not detectably transmitted by a prophage. Nature. 1983; 305:709–712.
3. Duthie ES, Lorenz LL. Staphylococcal coagulase; mode of action and antigenicity. Journal of general microbiology. 1952; 6:95–107.
4. Schneewind O, Mihaylova-Petkov D, Model P. Cell wall sorting signals in surface proteins of gram-positive bacteria. The EMBO journal. 1993; 12:4803–4811.
5. Yan J, Liu Y, Gao Y, Dong J, Mu C, Li D, Yang G. RNAIII suppresses the expression of LtaS via acting as an antisense RNA in Staphylococcus aureus. J Basic Microbiol. 2015; 55:255–261.
6. Murphy E, Huwyler L, de Freire Bastos Mdo C. Transposon Tn554: complete nucleotide sequence and isolation of transposition-defective and antibiotic-sensitive mutants. EMBO J. 1985; 4:3357–3365.
7. Boerlin P, Burnens AP, Frey J, Kuhnert P, Nicolet J. Molecular epidemiology and genetic linkage of macrolide and aminoglycoside resistance in Staphylococcus intermedius of canine origin. Vet Microbiol. 2001; 79:155–169.
8. Catchpole I, Thomas C, Davies A, Dyke KG. The nucleotide sequence of Staphylococcus aureus plasmid pT48 conferring inducible macrolide-lincosamide-streptogramin B resistance and comparison with similar plasmids expressing constitutive resistance. J Gen Microbiol. 1988; 134:697–709.
9. Ross JI, Eady EA, Cove JH, Cunliffe WJ, Baumberg S, Wootton JC. Inducible erythromycin resistance in staphylococci is encoded by a member of the ATP-binding transport super-gene family. Mol Microbiol. 1990; 4:1207–1214.
10. Luthje P, Schwarz S. Antimicrobial resistance of coagulase-negative staphylococci from bovine subclinical mastitis with particular reference to macrolide-lincosamide resistance phenotypes and genotypes. J Antimicrob Chemother. 2006; 57:966–969.
